# Comparison between liver resection and liver transplantation on outcomes in patients with solitary hepatocellular carcinoma meeting UNOS criteria: a population-based study of the SEER database

**DOI:** 10.18632/oncotarget.22134

**Published:** 2017-10-30

**Authors:** Anli Yang, Weiqiang Ju, Xiaopeng Yuan, Ming Han, Xiaoping Wang, Zhiyong Guo, Xiaoli Wei, Dongping Wang, Xiaofeng Zhu, Linwei Wu, Xiaoshun He

**Affiliations:** ^1^ Organ Transplant Center, The First Affiliated Hospital of Sun Yat-sen University, Guangzhou 510080, China; ^2^ Department of Medical Oncology, Sun Yat-sen University Cancer Center, State Key Laboratory of Oncology in South China, Collaborative Innovation Center for Cancer Medicine, Guangzhou 510060, China

**Keywords:** hepatocellular carcinoma, liver resection, liver transplantation, UNOS criteria

## Abstract

Liver resection (LR) and liver transplantation (LT) are potential curative treatment methods for early hepatocellular carcinoma (HCC). However, it is controversial which treatment is more beneficial to patients with solitary HCC meeting the United Network for Organ Sharing (UNOS) criteria (single lesion, diameter≤50mm, no vascular invasion, no extrahepatic metastasis). We retrieved patients with solitary HCC meeting UNOS criteria diagnosed between 2004-2013 from the Surveillance Epidemiology and End Results (SEER) database. Multivariate Cox proportional hazards regression models were used to evaluate the impact of surgery type (LR/LT) on overall survival (OS) and disease-specific survival (DSS) in both the whole study group and subgroups. Our analyses show that LT Patients had significantly superior OS (Adjusted *HR* (95% *CI*): 0.39 [0.26-0.59]) and DSS (Adjusted *HR* (95% *CI*): 0.19 [0.10-0.35]) than those receiving LR, although compared with the 288 patients receiving LR, the 258 patients receiving LT had younger age, smaller tumor size, and higher fibrosis score (*P*<0.001). Subgroup analyses identified significant interactions between surgery type (LR/LT) and gender (Male/Female) in both OS (*P*=0.02) and DSS (*P*=0.02). Male patients benefit more from LT compared with LR in both OS (Adjusted *HR* (95% *CI*): 0.29 [0.18-0.47]) and DSS (Adjusted *HR* (95% *CI*): 0.10 [0.05-0.21]), but there is no difference between patients receiving LT and LR in female patients. In conclusion, LT is associated with superior survival than LR in patients with solitary HCC meeting UNOS criteria. Moreover, male patients benefits more from LT than LR, while female patients do not show different outcomes between the two procedures.

## INTRODUCTION

As one of the leading causes of cancer-related mortality in the United States, hepatocellular carcinoma (HCC), along with intrahepatic cholangiocellular carcinoma, ranks fifth and eighth among men and women, respectively [1]. According to statistics based on Surveillance Epidemiology and End Results (SEER) registry data, the age adjusted incidence rate for HCC is at least 6 per 100,000 in the United States [2]. Even worse, the future burden of HCC is estimated to increase [2]. The detection rate of early-stage HCC has increased as a consequence of screening for high risk groups, such as hepatitis B virus-infected patients [3]. Correspondingly, a SEER registry data based analysis disclosed that the diagnosis of HCC with tumors ≤5.0 cm in diameter has significantly increased from 2000 to 2010, which has surpassed the diagnosis of HCC with large tumors [4]. The increased proportion of early HCC stresses the demand of choosing curative treatments appropriately specifically for this tumor type.

However, the optimal treatment for early-stage HCC has long been debated. Three treatment modalities have been considered to be potential options, including radiofrequency ablation (RFA), liver resection (LR) and liver transplantation (LT) [5]. The application of RFA is largely limited by tumor size [6]. RFA is reported to be inferior to LR in terms of recurrence-free survival and overall survival (OS). In addition, it is found to be worse than LT in terms of OS [7, 8]. Prospective randomized studies rarely focus on prognostic differences between patients receiving LR and LT. Related retrospective studies are mainly small in sample size and without uniform eligibility criteria for some important prognostic pathological characteristics (e.g., vascular invasion, lesion number and tumor size), resulting in controversial conclusions [9–11]. Even though LT offers an excellent curative chance for patients with specific criteria, options for patients are limited due to worldwide critical donor shortage [5, 12].

In view of the controversy, we compared outcomes of LR and LT among patients with solitary HCC meeting the United Network for Organ Sharing (UNOS) criteria (single lesion, diameter≤50mm, no vascular invasion, no extrahepatic metastasis). We also explored whether one treatment outperforms the other in subgroup examinations.

## RESULTS

### Demographic and clinicopathologic differences

Among 546 patients with solitary HCC meeting UNOS criteria, there were 52.7% (N=288) receiving LR and 47.3% (N=258) receiving LT. There were 74.9% male patients and 25.1% female patients (a male:female ratio of 3:1). The number of patients enrolled had been slightly increasing from 2004 to 2013. There were 55.3% patients with an elevated alpha fetal protein (AFP) level and the majority of patients (71.2%) showed severe liver fibrosis or cirrhosis (fibrosis score 5-6). Regarding ethnicity and marital status, the study population was dominated by white (62.8%) and married (64.7%) patients, respectively. The distribution of study population by basic demographic and clinicopathologic characteristics is shown in Table 1.

**Table 1:** Demographic and clinicopathologic differences between patients receiving liver resection and liver transplantation.

Characteristics	Total, no. (%)	Surgery type		*P* value
		LR, no. (%)	LT, no. (%)	
Age (years)				
Median (1^st^-3rd quartile)	59 (54-65)	62 (56-68)	57 (52-62)	
≤59	279 (51.1)	114 (39.6)	165 (64.0)	<0.001
>59	267 (48.9)	174 (60.4)	93 (36.0)	
Sex				0.46
Male	409 (74.9)	212 (73.6)	197 (76.4)	
Female	137 (25.1)	76 (26.4)	61 (23.6)	
Year of diagnosis				0.02
2004-2007	155 (28.4)	72 (25.0)	83 (32.2)	
2008-2010	181 (33.1)	89 (30.9)	92 (35.6)	
2011-2013	210 (38.5)	127 (44.1)	83 (32.2)	
Grade				0.01
III+IV	72 (13.2)	44 (15.3)	28 (10.9)	
II	290 (53.1)	162 (56.2)	128 (49.6)	
I	184 (33.7)	82 (28.5)	102 (39.5)	
Tumor size (mm)				<0.001
≤20	177 (32.4)	58 (20.1)	119 (46.1)	
21-30	166 (30.4)	97 (33.7)	69 (26.7)	
31-50	203 (37.2)	133 (46.2)	70 (27.2)	
AFP				0.09
Normal	244 (44.7)	119 (41.3)	125 (48.4)	
Elevated	302 (55.3)	169 (58.7)	133 (51.6)	
Fibrosis score				<0.001
0-4	157 (28.8)	133 (46.2)	24 (9.3)	
5-6	389 (71.2)	155 (53.8)	234 (90.7)	
Race				<0.001
American Indian/Alaska Native	7 (1.3)	4 (1.4)	3 (1.2)	
White	343 (62.8)	142 (49.3)	201 (77.9)	
Black	58 (10.6)	36 (12.5)	22 (8.5)	
Asian or Pacific Islander	138 (25.3)	106 (36.8)	32 (12.4)	
Marriage status				0.002
Widowed	28 (5.1)	23 (8.0)	5 (1.9)	
Married	353 (64.7)	173 (60.1)	180 (69.8)	
Others	165 (30.2)	92 (31.9)	73 (28.3)	

Abbreviation: LR, liver resection; LT, liver transplantation; AFP, alpha fetal protein.

Demographic and clinicopathololgic characteristics were compared between patients receiving LR and LT (Table 1). The median ages of patients receiving LR and LT were 62 and 57 years, respectively. Patients receiving LR were significantly older than those receiving LT (*P*<0.001). Male and female patients were evenly distributed between LR and LT group (*P*=0.46). From 2004 to 2013, the proportion of patients was increasing in LR group while decreasing in LT group (*P*=0.02). Patients receiving LR had significantly higher tumor grade (*P*=0.01) and larger tumor size (*P*<0.001), but lower fibrosis score (*P*<0.001). No remarkable difference was found in AFP level between LR and LT group (*P*=0.09). Significantly more white (*P*<0.001) and married (*P*=0.002) patients performed LT.

### Impact of OS by surgery type (LR/LT)

**Figure 1:**
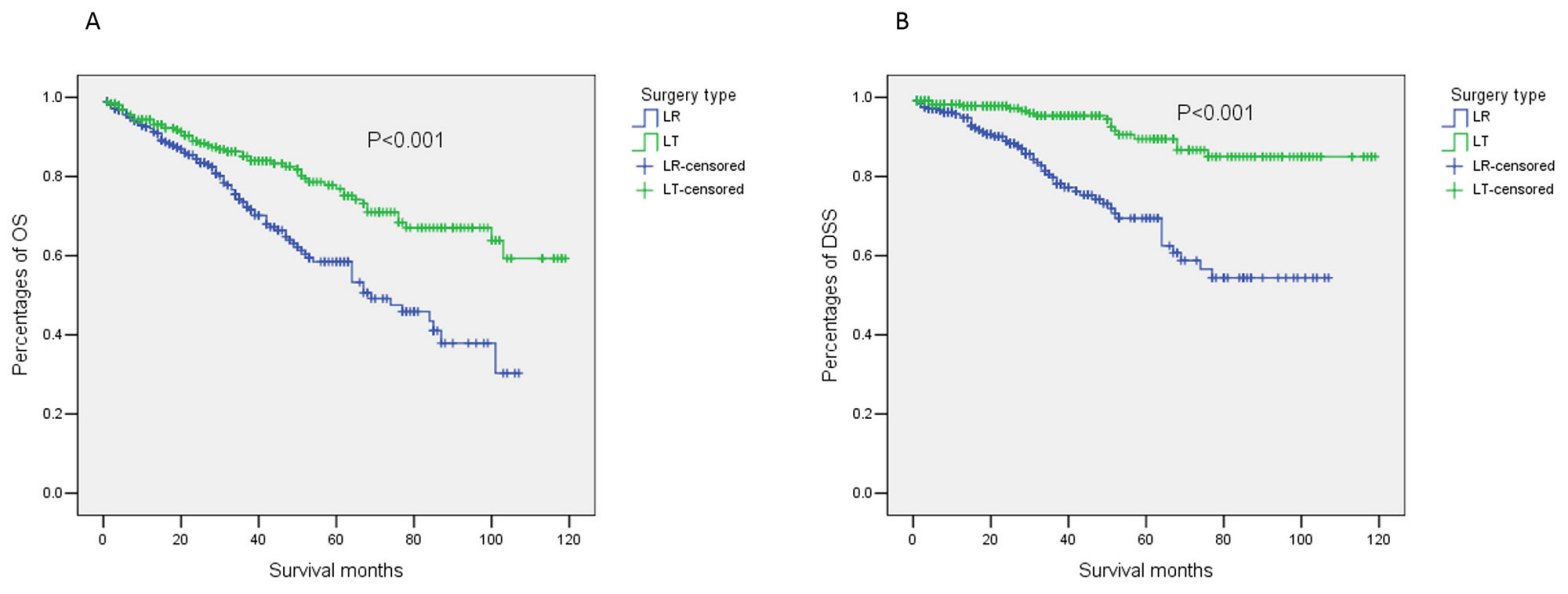
Outcomes of patients with solitary HCC meeting UNOS criteria stratified by surgery type (LR/LT) **(A)** OS; **(B)** DSS. Patients receiving LT had significantly superior OS to patients receiving LR (hazard ratio [*HR*] [95%*CI*]: 0.50 [0.35-0.70], *P*<0.001). Patients receiving LT also had significantly superior DSS to patients receiving LR (*HR* [95%*CI*]: 0.25 [0.15-0.43], *P*<0.001). Abbreviations: HCC, hepatocellular carcinoma; UNOS, the United Network for Organ Sharing; LR, liver resection; LT, liver transplantation; OS, overall survival; DSS, disease-sepecific survival.

The median OS of patients receiving LR was 69.0 months with the 95% confidence interval (*CI*) of 50.7-87.3 months, while the median OS of those receiving LT was not reached. The mean OS (95%*CI*) of patients receiving LR and LT was 67.6 (61.8-73.4) months and 91.9 (85.8-97.9) months, respectively. The OS of patients receiving LT was significantly superior to that of patients receiving LR based on univariate analysis (hazard ratio [*HR*] [95%*CI*]: 0.50 [0.35-0.70], *P*<0.001, Figure 1). We considered year of diagnosis, age at diagnosis, sex, tumor grade, tumor size, AFP level, fibrosis score, race, and marriage status for multivariable Cox regression model. Based on multivariate analysis, only surgery type (LR/LT) and fibrosis score (0-4/5-6) were significant and independent prognostic factors for OS. After being adjusted by year of diagnosis, age at diagnosis, sex, tumor grade, tumor size, AFP level, fibrosis score, race, and marriage status, patients receiving LT still had dramatically better OS with an adjusted *HR* (95%*CI*) of 0.39 (0.26-0.59) (*P* <0.001). Details of multivariate analyses are shown in Table 2.

**Table 2:** Multivariate Cox proportional hazards regression analyses for the impact of surgery type on OS and DSS.

Characteristics	OS		DSS		
	Adjusted *HR* (95% *CI*)	*P* value	No. (%)	Adjusted *HR* (95% *CI*)	*P* value
Surgery type (LR/LT)	0.39 (0.26-0.59)	<0.001	250 (50.9)/241 (49.1)	0.19 (0.10-0.35)	<0.001
Age (≤59/>59)	1.38 (0.97-1.97)	0.07	267 (54.4)/224 (45.6)	1.03 (0.62-1.71)	0.91
Sex (Male/Female)	1.18 (0.80-1.74)	0.41	376 (76.6)/115 (23.4)	1.21 (0.70-2.12)	0.50
Year of diagnosis		0.30			0.83
2004-2007	1 (Reference)		136 (27.7)	1 (Reference)	
2008-2010	0.84 (0.56-1.25)	0.39	171 (34.8)	0.88 (0.48-1.60)	0.66
2011-2013	0.64 (0.36-1.14)	0.13	184 (37.5)	1.07 (0.48-2.41)	0.87
Grade		0.56			0.08
III+IV	1 (Reference)		64 (13.0)	1 (Reference)	
II	0.76 (0.45-1.29)	0.32	258 (52.5)	0.47 (0.24-0.91)	0.03
I	0.75 (0.43-1.31)	0.32	169 (34.4)	0.54 (0.26-1.10)	0.09
Tumor size (mm)		0.57			0.31
≤20	1 (Reference)		160 (32.6)	1 (Reference)	
21-30	1.23 (0.80-1.90)	0.34	149 (30.3)	1.44 (0.74-2.82)	0.28
31-50	1.04 (0.67-1.60)	0.87	182 (37.1)	1.65 (0.87-3.12)	0.12
AFP (Normal/ Elevated)	0.77 (0.54-1.09)	0.14	214 (43.6)/277 (56.4)	0.72 (0.43-1.20)	0.20
Fibrosis score (0-4/5-6)	0.54 (0.36-0.82)	0.003	133 (27.1)/358 (72.9)	0.37 (0.21-0.6)	0.001
Race			0.12		0.53
American Indian/Alaska Native	1 (Reference)		5 (1.0)	1 (Reference)	
White	0.38 (0.11-1.26)	0.11	307 (62.5)	0.53 (0.07-4.09)	0.55
Black	0.42 (0.12-1.48)	0.17	53 (10.8)	0.69 (0.08-5.71)	0.73
Asian or Pacific Islander	0.27 (0.08-0.92)	0.04	126 (25.6)	0.40 (0.05-3.24)	0.39
Marriage status			0.98		0.69
Widowed	1 (Reference)		19 (3.9)	1 (Reference)	
Married	1.04 (0.50-2.16)	0.92	322 (65.6)	1.27 (0.42-3.87)	0.68
Others	1.00 (0.47-2.11)	0.99	150 (30.5)	1.00 (0.33-3.07)	0.99

Abbreviation: OS, overall survival; DSS, disease-specific survival; *HR*, hazard ratio; *CI*, confidence interval; LR, liver resection; LT, liver transplantation; AFP, alpha fetal protein.

### Impact of disease-specific survival (DSS) by surgery type (LR/LT)

To reduce the impact of comorbidities on the survival differences between patients receiving LR and LT, we performed univariate and multivariate Cox proportional hazard regression analyses for DSS. The median DSS (95%*CI*) of both groups was not reached, and the mean DSS (95%) was 77.5 (71.1-83.9) months in patients receiving LR and 108.2 (103.5-112.9) months in those receiving LT. The DSS in patients receiving LT was likewise significantly superior to those receiving LR in univariate analysis (*HR* [95%*CI*]: 0.25 [0.15-0.43], *P*<0.001, Figure 1). The results of multivariate analysis for DSS were similar to those for OS. Patients receiving LT had a prominently superior DSS compared with patients receiving LR after being adjusted by the aforementioned confounding factors (adjusted *HR* [95%*CI*]: 0.19 [0.10-0.35], *P*<0.001, Table 2).

### Sex based disparities in the impact of surgery type on OS and DSS

**Figure 2:**
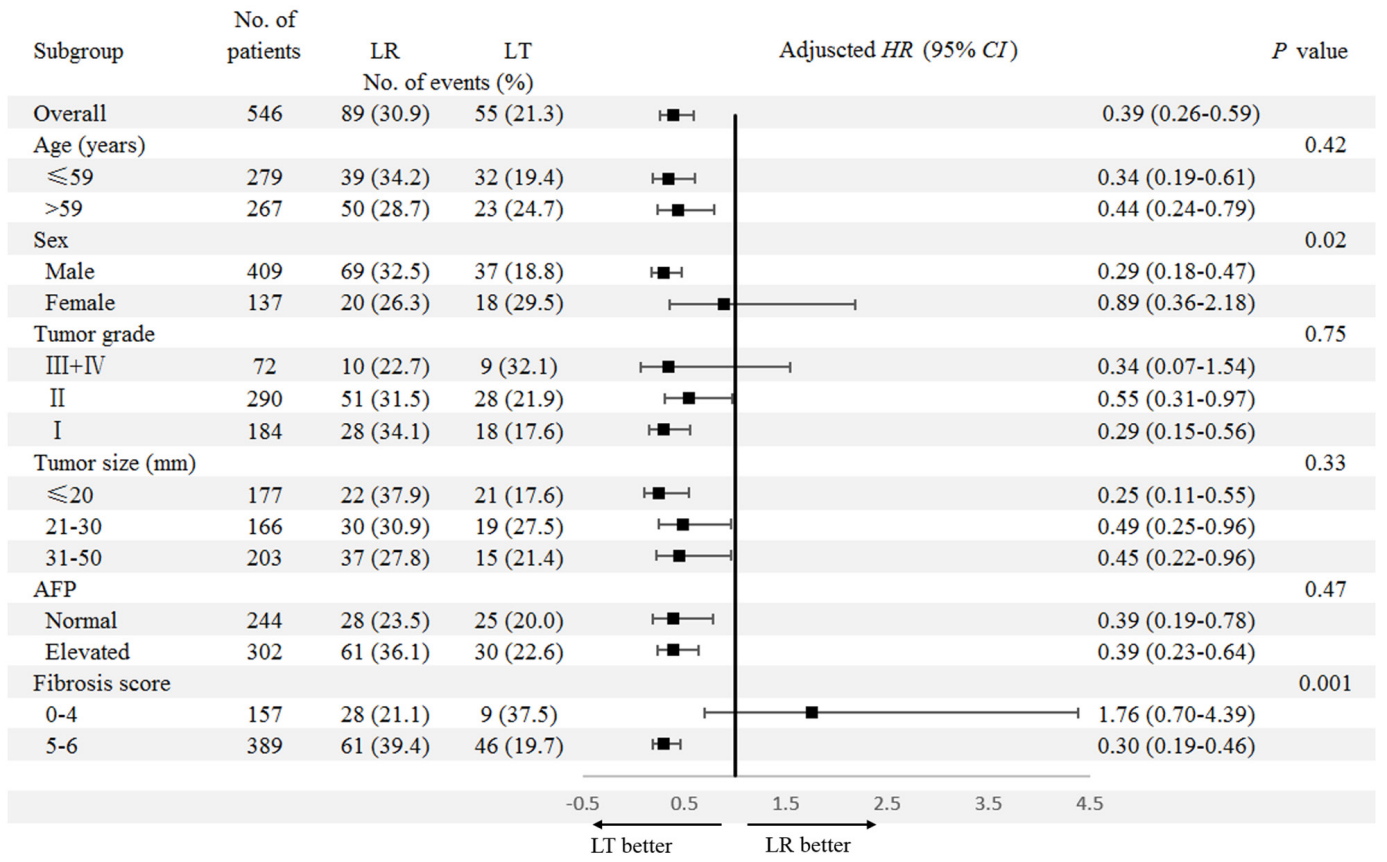
Subgroup analyses for the impact of surgery type (LR/LT) on OS Comparisons of the OS between patients receiving LR and LT were conducted in subgroups stratified by clinicopathologic factors. LR group was used as the reference for adjusted *HR* (95%*CI*). The factors considered for the multivariable Cox regression model included year of diagnosis, age at diagnosis, sex, tumor grade, tumor size, AFP level, fibrosis score, race, and marriage status. Abbreviations: OS, overall survival; LR, liver resection; LT, liver transplantation; *HR*, hazard ratio; *CI*, confidence interval; AFP, alpha fetal protein.

**Figure 3:**
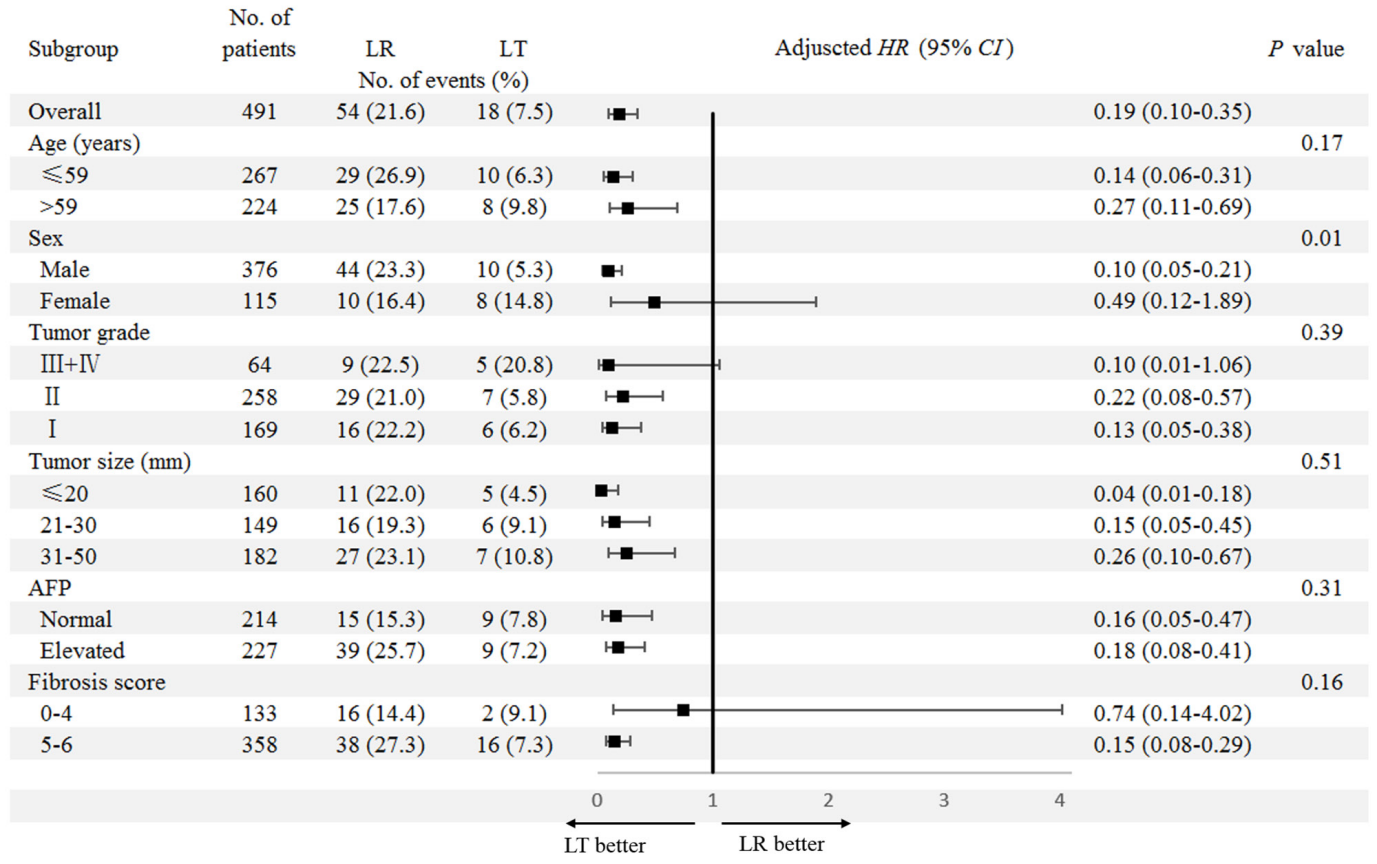
Subgroup analyses for the impact of surgery type (LR/LT) on DSS Comparisons between the DSS for patients receiving LR and LT were conducted in subgroups stratified by clinicopathologic factors. LR was used as the reference group for adjusted *HR* (95%*CI*). The factors considered for the multivariable Cox regression model included year of diagnosis, age at diagnosis, sex, tumor grade, tumor size, AFP level, fibrosis score, race, and marriage status. Abbreviations: DSS, disease-specific survival; LR, liver resection; LT, liver transplantation; *HR*, hazard ratio; *CI*, confidence interval; AFP, alpha fetal protein.

**Figure 4:**
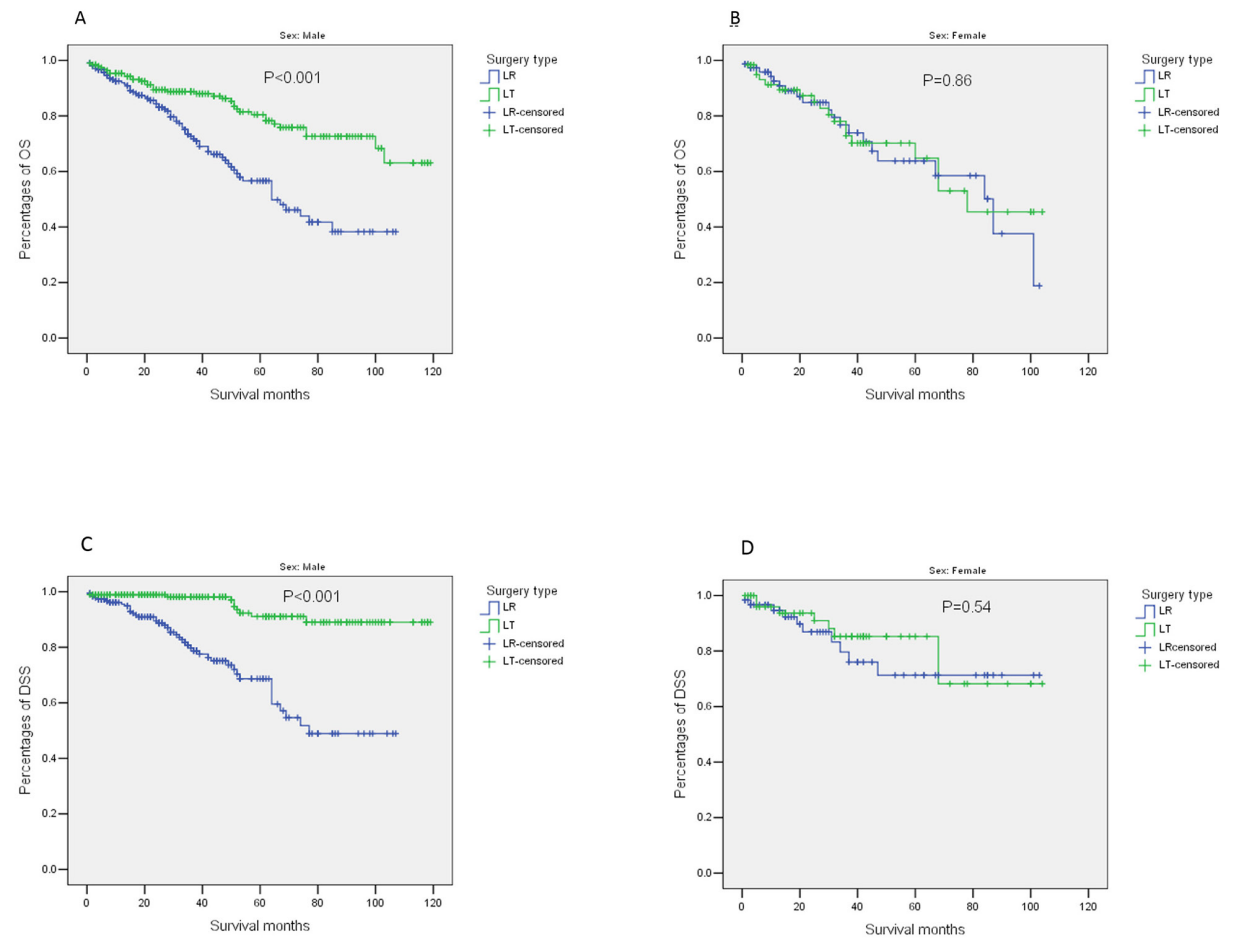
Outcomes of patients stratified by surgery type (LR/LT) and sex (male/female) **(A)** OS, Male; **(B)** OS, Female; **(C)** DSS, Male; **(D)**, DSS, Female. Abbreviations: LR, liver resection; LT, liver transplantation; OS, overall survival; DSS, disease-free survival.

Next, we examined survival differences between patients receiving LR and LT in subgroups (Figure 2 for OS and Figure 3 for DSS). Consistently superior OS for patients receiving LT was identified in subgroups stratified by age (≤59/>59 years), tumor grade (III+IV/II/I), tumor size (≤20/21-30/31-50mm), and AFP level (Normal/Elevated) from univariate and multivariate analyses. It is worth noting that there was a significant interaction between surgery type (LR/LT) and sex (Male/Female) (*P*_interaction_=0.02). A significantly superior OS for patients receiving LT (mean OS [95%*CI*]: 95.8 [89.4-102.3]) compared to those receiving LR (mean OS [95%*CI*]: 67.0 [60.2-73.7]) was identified in males (adjusted *HR* [95%*CI*]: 0.29 [0.18-0.47], *P*<0.001). While no difference in OS was found between patients receiving LT (mean OS [95%*CI*]: 71.0 [9.5-82.6]) and LR (mean OS [95%*CI*]: 95.8 [89.4-102.3]) in females (adjusted *HR* [95%*CI*]: 0.89 [0.36-2.18], *P*=0.79) (Figure 4). In addition, there was no difference in OS between patients receiving LT (mean OS [95%*CI*]: 72.0 [53.6-90.4]) and LR (mean OS [95%*CI*]: 79.2 [71.2-87.2]) in those with normal to moderate liver fibrosis (fibrosis score 0-4) (adjusted *HR* [95%*CI*]: 1.76 [0.70-4.39], *P*=0.23). In contrast, in patients with severe liver fibrosis or cirrhosis (fibrosis score 5-6), those receiving LT (mean OS [95%*CI*]: 93.8 [87.5-100.0]) had significant superior OS to those receiving LR (mean OS [95%*CI*]: 57.2 [49.3-65.0]). A significant interaction was also identified between surgery type (LR/LT) and fibrosis score (0-4/5-6) (*P*_interaction_=0.001) (Supplementary Figure 1). Details of the subgroup analyses for differences in OS between patients receiving LR and LT are shown in Figure 2.

In subgroup analyses for DSS, only sex (Male/Female) was identified to have a significant interaction with surgery type (LR/LT) on DSS (*P*_interaction_=0.01). In male patients, there was a notably superior DSS in those receiving LT (mean DSS [95%*CI*]: 111.4 [107.0-115.9]) to those receiving LR (mean DSS [95%*CI*]: 75.3 [68.0-82.7]) (adjusted *HR* [95%*CI*]: 0.10 [0.05-0.21], *P*<0.001). While no difference was found in female patients (Figure 4). The differences in DSS stratified by surgery type (LR/LT) and fibrosis score (0-4/5-6) are shown in Supplementary Figure 1. When the impact of comorbidities was excluded in the analyses of cancer-specific outcomes, the interaction between fibrosis score (0-4/5-6) and surgery type (LR/LT) was reduced, which became non-significant for DSS (*P*_interaction_=0.16, Figure 3).

## DISCUSSION

To our knowledge, this is the first study to compare the outcomes of LR and LT in patients with solitary HCC who met UNOS criteria. The UNOS criteria specifies that patients who met the criteria should be candidates for LT. However, there has been no strong clinical evidence supporting this proposal. No prospective randomized studies has been conducted to compare the effectiveness of LR and LT in this type of patients. Though several retrospective studies focused on this issue, their results were controversial due to different eligibility criteria, especially in tumor size, lesion number and vascular invasion [9–11, 13, 14]. According to a meta-analysis comparing outcomes of LR and LT in patients with early HCC, LT group had a survival advantage in certain settings. Nevertheless, the conclusion was not solid enough due to the limited number of studies included [15]. Therefore, we brought a relatively large number of strictly screened patients, who were diagnosed with solitary HCC meeting UNOS criteria into the current study in order to understand whether patients receiving LT could get survival advantage compared with those receiving LR.

In the first-line treatment of early HCC, the selection of three main potential curative therapies (RFA, LR and LT) had been intensively investigated [5, 8, 16–18]. Factors affecting selection included tumor characteristics (i.e., location, size, lesion number, vascular invasion) and patient status (i.e., performance status, liver function, portal hypertension, Child-Pugh score). Additionally, some external factors also had significant impact on treatment selection. One of the most notable ones was the contradiction between increasing demand of LT and the scarcity of organ donors [19]. Taking both urgency and cost-effectiveness into consideration, LT used to be recommended as a second-line treatment for very early HCC [5, 20–23]. But actually, as demonstrated by reports from other centers and the current study [8, 10], LT provided more survival benefit for very early HCC than LR and RFA. The key to resolve the issue of contradiction between supply and demand may be to identify the subgroups of patients likely to benefit the most from LT, so that we could make better use of the limited resource of graft.

In the subgroup analyses of our study, significant interactions between gender and surgery type were demonstrated in both OS and DSS. In patients with solitary HCC meeting UNOS criteria, we found that male patients benefit more from LT compared with LR, but female patients do not. One potential reason for this discrepancy may be gender disparity in immunity. Females had been described as more “immunogenic” than males [24], while men were more inclined to achieve immune tolerance after transplantation [25]. Similarly, female gender was reported to be a risk factor for graft loss in patients transplanted for hepatitis C virus (HCV)-related cirrhosis [26, 27]. Female patients also had lower rate of sustained response and higher rate of relapse in anti-HCV treatment compared with male patients [28]. In a prospective cohort study of patients receiving LT in Italy, five-year graft survival was significantly lower in HCV-positive patients and recipient female gender was an independent indicator of graft loss, but all additional mortality in females was found to be attributable to severe HCV recurrence [26]. Additionally, an interaction between female gender and HCV infection was identified in risk of chronic renal failure after LT [29]. Thus, it seemed that there could be negative interactions between female gender and HCV infection in patients receiving LT. However, this couldn’t be analyzed in the current study due to lack of etiology data of HCC in SEER database. Moreover, according to a study on the impact of gender on survival of patients with HCC, female patients had significantly superior outcomes compared with male patients in those receiving LR, but not in those receiving LT [30]. This finding is consistent with gender disparity identified in our study. However, the underlying mechanism is still unclear. It is very important to verify the gender disparity in the impact of surgery types on outcomes of patients with early HCC before it can be translated into clinical practice.

LT was considered to be the optimal modality for treatment of HCC, because it extirpated both the tumor and the underlying liver disease. In the current study, LT was found to be a better option for patients with severe fibrosis or cirrhosis (fibrosis score 5-6), but LT was not superior to LR for patients with none to moderate fibrosis (fibrosis score 0-4). This is in accordance with previous studies [31–33]. When the impact of comorbidities was excluded in the analyses for DSS, the interaction between fibrosis score (0-4/5-6) and surgery type (LR/LT) was diminished. LT could cure the underlying liver diseases, thus remitting the relevant comorbidities, possibly making it superior to LR in OS. In other words, our study supports the principle of LT precedence in HCC patient with cirrhosis. However, the cause of fibrosis or liver cirrhosis was not analyzed in the current study due to lack of relevant data in SEER database.

The retrospective nature is one of the limitations of this study. However, there has been no prospective randomized clinical trials to-date on this issue. Additionally, because some important prognostic factors were not recorded in SEER database, such as comorbidities, liver function, physical status, Child-Pugh classifications, Model for End-Stage Liver Disease (MELD) score, and etiology factors, we adopted DSS as a secondary outcome to isolate the impact of surgery type on HCC-specific outcomes. There was no information on HCC recurrence in SEER database, thus we couldn’t analyze the therapy of HCC recurrence and re-resection rate in the current study. Furthermore, only those receiving LR or LT were included for analyses, some complicated cases, such as liver resection as bridging therapy to transplantation, were not analyzed in the current study. These issues might be clarified in future investigations with more detailed information. In conclusion, with a relatively large study population, our study demonstrated that LT was superior to LR on prognosis of patients with solitary HCC meeting UNOS criteria. Moreover, male patients and patients with severe fibrosis or cirrhosis were possibly optimal subgroups who could benefit the most from LT.

## MATERIALS AND METHODS

### Ethics statement

This study was deemed exempt from institutional review board approval by The First Affiliated Hospital of Sun Yat-sen University and Sun Yat-sen University Cancer Center, thus informed consent was waived. This study was conducted in accordance with the ethical standards of the World Medical Association Declaration of Helsinki.

### Database and patient selection

The SEER database, the largest publicly available cancer dataset, is a population-based cancer registry across several disparate geographic regions in the United States. The SEER research data include cancer incidence and demographic information tabulated by age, sex, race/ethnicity, year of diagnosis and geographic region. In addition, some clinicopathologic characteristics are also included. The SEER research database from 1973 to 2013 (Nov 2015 Submission) was retrieved for the current study.

**Figure 5:**
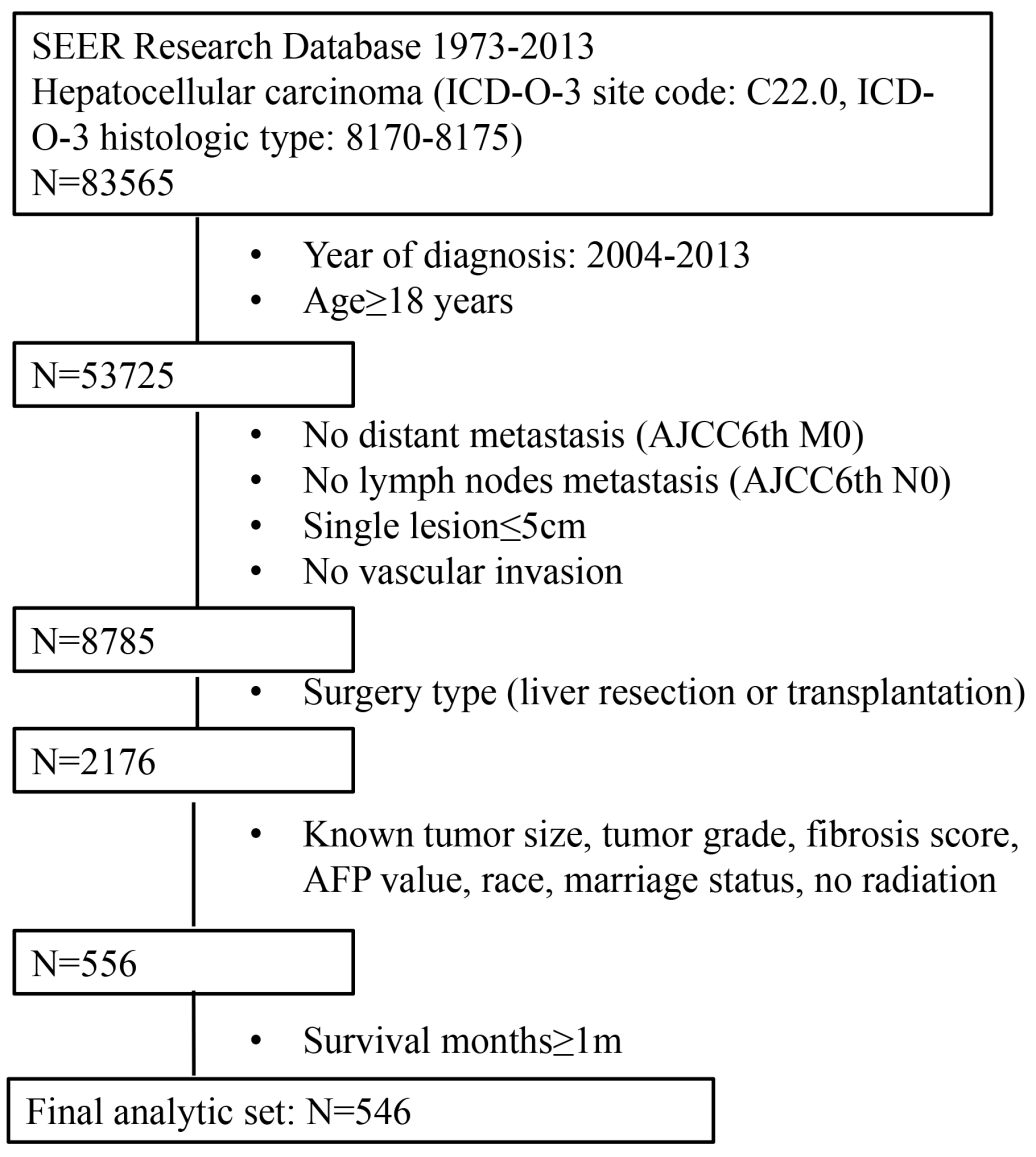
The flow chart for selection of study population Abbreviations: SEER, Surveillance, Epidemiology, and End Results; ICD-O-3, international classification of diseases for oncology, 3^rd^ edition; AJCC, American Joint Committee on Cancer; AFP, alpha fetal protein.

There were 83,565 patients diagnosed with HCC retrieved from SEER database. We then enrolled patients not younger than 18 years old and diagnosed from 2004 to 2013. Furthermore, only those with solitary tumors, meeting UNOS criteria and receiving LR or LT were included for analysis. Patients lacking important demographic and clinicopathologic information were excluded. Patients receiving radiation or with incomplete survival data were also excluded. Detailed inclusion and exclusion criteria and the numbers of patients are shown in Figure 5. At the end, 546 patients were included in this study.

### Definitions

The diagnosis of HCC was identified with international classification of diseases for oncology, 3^rd^ edition [ICD-O-3] site code C22.0 and histologic type code 8170-8175. The lesion number and the status of vascular invasion were identified using the codes of CS extension (2004+) (http://web2.facs.org/cstage0205/liver/Liver_bbc.html). The type of surgery was converted by the codes of RX Summ-Surg Prim Site (1998+): LR: 20 to 25, 30, 36, 37, 50, 51, and 52; LT: 61. Patients receiving LR or LT plus other therapies were excluded. Detailed definitions of each variable are shown in Supplementary Table 1.

### Outcomes

The primary outcome was OS, which was defined as the time interval between the diagnosis of HCC and the death of any cause. Live patients were censored at the last recorded follow-up time. Additionally, because patients with HCC were frequently presented with life-threatening comorbidities, DSS was used as a secondary outcome, which was defined as time interval between the diagnosis of HCC and the death attributed to HCC. Deaths from other causes were calculated as censored cases.

### Statistical analyses

The statistical analyses were performed with SPSS for Windows V.13.0. (SPSS Inc., Chicago, IL, USA). The demographic and clinicopathologic differences between patients receiving LR and LT were evaluated with chi-square test or Kruskal-Wallis H test based on the type of the data and comparisons. Survival curves were plotted by the Kaplan-Meier method and compared using the log-rank test. Univariate and multivariate Cox proportional hazards regression models were used to evaluate the impact of surgery type (LR/LT) on OS and DSS. Multivariate Cox proportional hazards regression analyses were adjusted by year of diagnosis, age at diagnosis, sex, tumor grade, tumor size, AFP level, fibrosis score, race, and marriage status. A likelihood ratio test was applied to examine the interactions between surgery type (LR/LT) and clinicopathologic characteristics on OS and DSS.

## SUPPLEMENTARY MATERIALS FIGURE AND TABLE


