# Supplementary material for: Comparison between liver resection and liver transplantation on outcomes in patients with solitary hepatocellular carcinoma meeting UNOS criteria: a population-based study of the SEER database
Source: Oncotarget. 2017 Oct 30;8(57):97428–38. doi: 10.18632/oncotarget.22134 (PMC5722574; doi:10.18632/oncotarget.22134)
Supplement: Supplementary file 1 [file oncotarget-08-97428-s001.pdf]

Comparison between liver resection and liver transplantation on outcomes in patients with solitary hepatocellular carcinoma meeting UNOS criteria: a population-based study of the SEER database

SUPPLEMENTARY MATERIALS

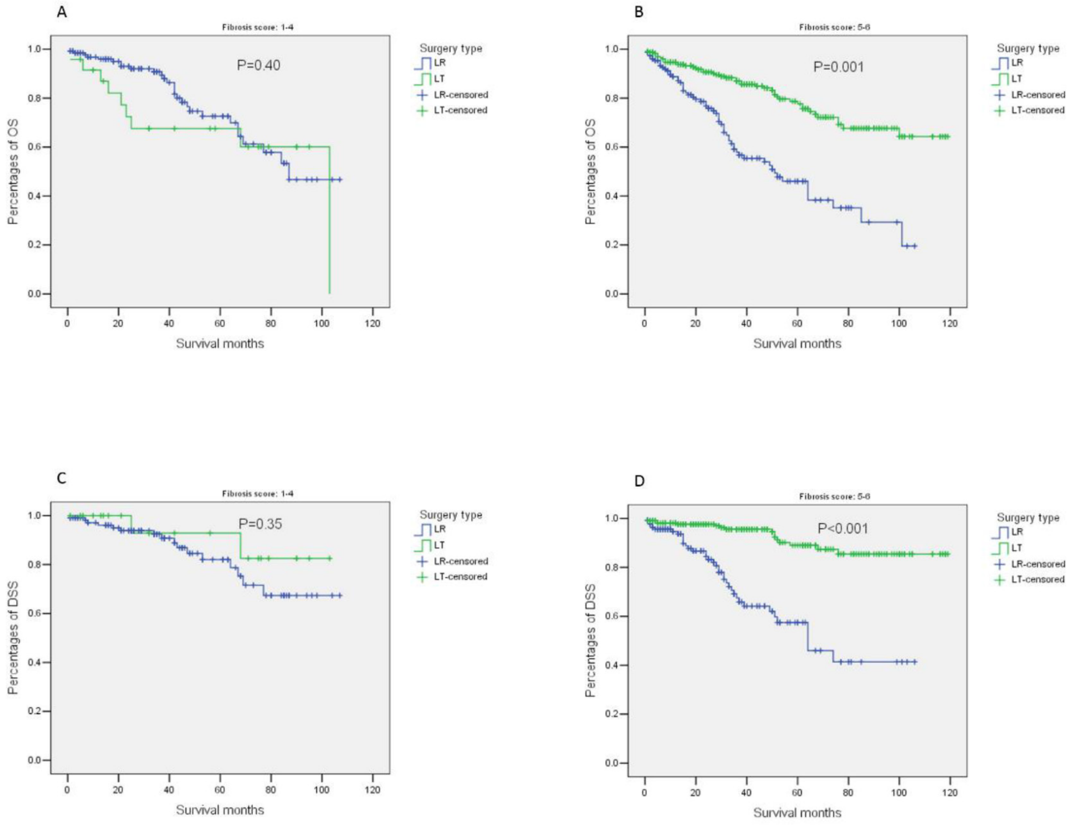

Supplementary Figure 1: Outcomes of patients stratified by surgery type (LR/LT) and fibrosis score (0-4/5-6). (A) OS, Fibrosis score 0-4; **(B)** OS, Fibrosis score 5-6; **(C)** DSS, Fibrosis score 0-4; **(D)**, DSS, Fibrosis score 5-6. Abbreviations: LR, liver resection; LT, liver transplantation; OS, overall survival; DSS, disease-free survival.

Supplementary Table 1. Variable categories and Codes

Variables	Code name	Categories	Codes
Lesion number	CS extension (2004+)	Single lesion	100, 150, 250, 270, 350, 370, 380
		More than one lesion	300, 390, 400, 420, 440, 600, 770
Vascular invasion	CS extension (2004+)	Negative	100, 150, 250, 270, 390
		Positive	350, 370, 380, 400, 630, 635, 638, 660
Tumor size (mm)	CS tumor size (2004+)	1-20	1-20, 991, 992
		21-30	21-30, 993
		31-50	31-50, 994, 995
		>50	51-989, 996
Surgery type	RX Summ-Surg Prim Site (1998+)	LR	20-25, 30, 36, 37, 50, 51, 52
		LT	61

Abbreviation: CS, collaborative stage; LR, liver resection; LT, liver transplantation;
